# Service-oriented Device Connectivity interface for a situation recognition system in the OR

**DOI:** 10.1007/s11548-022-02666-4

**Published:** 2022-05-20

**Authors:** Denise Junger, Patrick Beyersdorffer, Christian Kücherer, Oliver Burgert

**Affiliations:** School of Informatics, Research Group Computer Assisted Medicine (CaMed), Reutlingen University, Reutlingen, Germany

**Keywords:** Context awareness, Situation recognition system, Context-aware system, SDC, OR-Pad, Intraoperative area

## Abstract

**Purpose:**

Context awareness in the operating room (OR) is important to realize targeted assistance to support actors during surgery. A situation recognition system (SRS) is used to interpret intraoperative events and derive an intraoperative situation from these. To achieve a modular system architecture, it is desirable to de-couple the SRS from other system components. This leads to the need of an interface between such an SRS and context-aware systems (CAS). This work aims to provide an open standardized interface to enable loose coupling of the SRS with varying CAS to allow vendor-independent device orchestrations.

**Methods:**

A requirements analysis investigated limiting factors that currently prevent the integration of CAS in today's ORs. These elicited requirements enabled the selection of a suitable base architecture. We examined how to specify this architecture with the constraints of an interoperability standard. The resulting middleware was integrated into a prototypic SRS and our system for intraoperative support, the *OR-Pad*, as exemplary CAS for evaluating whether our solution can enable context-aware assistance during simulated orthopedical interventions.

**Results:**

The emerging *Service-oriented Device Connectivity* (SDC) standard series was selected to specify and implement a middleware for providing the interpreted contextual information while the SRS and CAS are loosely coupled. The results were verified within a proof of concept study using the *OR-Pad* demonstration scenario. The fulfillment of the CAS’ requirements to act context-aware, conformity to the SDC standard series, and the effort for integrating the middleware in individual systems were evaluated. The semantically unambiguous encoding of contextual information depends on the further standardization process of the SDC nomenclature. The discussion of the validity of these results proved the applicability and transferability of the middleware.

**Conclusion:**

The specified and implemented SDC-based middleware shows the feasibility of loose coupling an SRS with unknown CAS to realize context-aware assistance in the OR.

## Introduction

Context-aware systems (CAS) within the interconnected operating room (OR) are an emerging research topic [1]. CAS provide surgeons with intervention-specific functionality depending on the current intraoperative situation. To achieve this, contextual information, e.g., device parameters, used instruments, etc., needs to be captured and analyzed. One of our applications for CAS is the *OR-Pad* system that addresses the improvement in the information flow for the surgeon within the perioperative area [2]: The system consists of a pre- and postoperative as well as intraoperative mode. Clinical information can be preselected preoperatively for display in specific surgical phases. Intraoperatively, this information shall be displayed automatically at the right time. In addition, the remaining surgery duration (RSD) is provided and new information (e.g., notes) can be added. After surgery, all information is available for postoperative usage. To enable context-aware provision, an extern situation recognition system (SRS) should be connected which provides the actual surgical phase and the RSD during surgery.

Junger et al. [3] present a concept and basic framework prototype for an SRS in the OR. The aim of the system is the flexible and intervention-independent recognition of the actual situation in the OR. The estimated contextual information, like the surgical phase or RSD, shall then be provided to CAS. The SRS acts as a self-contained system that collects contextual information from the OR and serves different CAS. It should be unimportant for the SRS what the CAS are designed for. Possible use cases are filtering information automatically [4], providing pre-assigned information [2, 5, 6], selecting and controlling devices [7, 8], or minimizing adverse events [9, 10]. With a uniform SRS, CAS can act context-aware without bothering about implementing own recognition approaches, preventing them to get too complex, and all having the same data basis. This may open up new research projects focusing on context-aware support. To achieve such a flexible system architecture, an interface is needed for providing the collected contextual information of the SRS to CAS appropriately.

A standardized medical protocol is necessary for this interface to support unknown CAS in a non-proprietary vendor-independent way. In a preliminary study, we investigated existing interface standards for their applicability in the context of CAS. The *Health Level Seven Version 2* [11], *Fast Healthcare Interoperability Resources* [12]*,* and *IEEE 11073 Service-oriented Device Connectivity* (SDC) standard series [13] were compared, as these were considered in the *Integrating the Healthcare Enterprise* (IHE) profile for *Service-oriented Device Point-of-care Interoperability* (SDPi) [14] to be possibly suitable for the interoperable networking of medical devices. In addition to this SDPi recommendation, *Digital Imaging and Communications in Medicine* (DICOM) [15] was included in the study since it is also used in research projects [16–18] as well as in clinical routine for computer-assisted planning and assistance. The DICOM *Unified Procedure Step Service-Object-Pair* [15] allows the modeling and provision of temporal and content differentiated contextual information. The *Service-oriented Medical Device Architecture* (SOMDA) of the SDC standards has also proven to be potentially suitable for providing contextual information. However, SDC *Device Specializations* [19] for an SRS and corresponding CAS do not currently exist and are not planned. The growing participation of research and industry in SDC-based medical device networking [20] and the possibility to achieve vendor-independent interoperability [13] justifies investigating how the SDC standards can be used to specify an interface for contextual information.

**Fig. 1:**
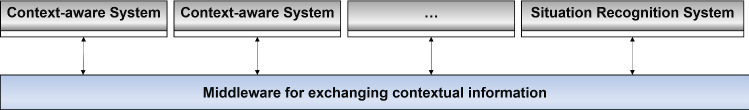
Desired system architecture for loose coupling an SRS with unknown CAS

System architectures for a context-aware OR already exist [21–23], but do not support loose coupling of unknown CAS and an SRS which is essential to allow vendor- and device-independent orchestrations. In this work, we specify and implement an SDC interface for the standardized communication between an intervention-independent SRS and unknown CAS to enable context-aware assistance in the OR (Fig. 1). Intraoperative device connectivity can also be achieved using the *Open Integrated Clinical Environment* [24] or the *Robot Operating System* [25]. The middleware in these architectures provides comprehensive functionality to heterogeneous network nodes. In contrast, our architecture uses a remote procedure call (RPC)-based middleware encapsulating the SDC complexity for exchanging contextual information with simple function calls. For evaluation, our implementation is integrated into the SRS of [3] and the context-aware *OR-Pad* of [2] providing and receiving contextual information by interacting with the middleware during seven simulated orthopedical interventions.

## Methods

### Requirements analysis

The system idea is concretized by system goals (SG) depicted in Table 1. The requirements for specifying these system goals were extracted from six published articles on prototypic CAS for surgery [1, 7, 13, 21, 23, 26]. Integration of CAS in clinical routine is still pending [1]. Limiting factors that prevent the integration were derived from the publications and formulated as requirements (Table 1). SG1 requires interoperability of the interface to realize the system idea of providing unknown CAS with contextual information. SG2 and SG3 address adaptation to varying medical device orchestrations or different context-aware use cases in an OR, e.g., devices with varying computing capabilities or different types and granularities of contextual information available. SG4 requires reliable risk management.

**Table 1:** System architecture goals and requirements

ID	System goals	Requirements			
SG1	Vendor-independent exchange of contextual information	R01	The interface shall be based on a standardized syntax	Interoperability	[13]
		R02	The interface shall be based on a standardized semantic	Interoperability	[13]
SG2	Device-independent exchange of contextual information	R03	Contextual information must be continuously accessible by the CAS	Interaction	[23]
		R04	Resource-constrained devices must be able to act context-sensitively	Interaction	[23]
SG3	Configurable exchange of contextual information	R05	CAS must be able to specifically query contextual information entities	Interaction	[21]
		R06	CAS must be able to subscribe to specifically contextual information entities	Interaction	[23]
		R07	Contextual information can have a varying level of granularity	Interaction	[26]
SG4	Controllable exchange of contextual information	R08	Contextual information must be provided simultaneously to multiple CAS	Risk management	[7]
		R09	The interface is not responsible for triggered context-aware functionality	Risk management	[21]
		R10	Changing contextual information shall be provided atomically	Risk management	[21]
		R11	The interface shall be fault-tolerant regarding missing contextual information	Risk management	[1]

### Selection of a base architecture

Currently, no proven design patterns exist for context-aware ORs [1]. Instead, empirical base architectures were compared.

Context awareness in the system architecture according to [21] is based on rules that follow a strict event–condition–action pattern and are evaluated by the CAS themselves. If a defined event occurs and the stored conditions are fulfilled, an action is automatically triggered. For risk management, the rules are tested in a separate research environment using recorded intraoperative data. For rule creation, it must be known which devices are involved and which intervention is performed. A central component for analyzing and providing contextual information is missing. Estimations about the intraoperative situation purely depend on atomic rules. Multisensory interpretations to provide complex contextual information, e.g., superordinate surgical phases, are not possible. This leads to the decision against using this architecture.

Neumann et al. implemented surgical workflow management using the *BPMN*^*SIX*^ extension [27] for context-aware remote control and orchestration of medical devices [22]. The orchestration of the CAS and the context-aware functions to be triggered are formalized in the surgical process model (SPM) using the *Business Process Model and Notation* (BPMN) [28], where the *Surgical Intervention Extension* (SIX) allows modeling of intraoperative entities. The required devices in the OR network are discovered using SDC mechanisms based on their offered functions. These functions are automatically started by the workflow management system (WfMS) during the execution of the SPM at the appropriate point of time using the respective SDC *Service and Control Object* (SCO) [29]. This system architecture enables context awareness in the OR exclusively by the WfMS and depends on the SPM for a specific intervention with stored CAS functions to be triggered. The desired loose coupling is not possible using this approach.

Franke and Neumuth developed three message exchange patterns to provide different devices with contextual information [23]. Multi-perspective information [30] is collected by a central *Workflow Information System* (WIS) [31] using intraoperative sensors. Medical devices receive contextual information from this WIS for the adaptation to the current intraoperative situation. Resource-constrained devices cannot process this information themselves, so the WIS provides a service to register rules that specify when and which kind of contextual information shall be sent. Device orchestrations are configured as profiles in a *Configuration Component*. Surgeons can select the appropriate profile for an intervention. The associated rules are then stored in the WIS, thus enabling context-aware behavior for multiple devices. The authors emphasize that the medical devices are responsible for the functions triggered based on the contextual information received, and potential hazards must therefore be considered in the respective risk management.

The components of the latter approach harmonize with our desired system architecture from Fig. 1. The message exchange patterns can fulfill the requirements and enable providing contextual information. Thus, we selected this approach as base architecture. However, rules for notifying the CAS shall not be stored in the SRS to obtain the loose coupling between SRS and CAS. The *Configuration Component* could be used to communicate with the SRS on behalf of resource-constrained devices and control them in a context-sensitive way. The message exchange patterns have been implemented by the authors with the *Open Surgical Platform*, a precursor of the SDC standards. How the contextual information is encoded and via which services it is exchanged is not further specified in [23]. We specified this base architecture with the current version of the SDC standards and embed the message exchange patterns in a middleware.

### Specification and implementation of the middleware

The components and their interactions derived from the selected base architecture [23] were specified by the constraints of the SDC standard series, namely by the IEEE 11073 standard parts [29] (*Domain Information and Service Model*), [32] (*Nomenclature*), [33] (*Communication Profile for Web Services*), and [34] (*Protocol Binding*). The *sdcLib* programming library [35] was used for implementation since this library has been successfully applied in other research projects [21, 36–38]. The standardized SDC data transfer of contextual information with associated discovery and security aspects shall be encapsulated by our RPC-based middleware. For evaluation, a prototype was implemented and integrated into the SRS of [3] and context-aware *OR-Pad* of [2] in the research OR of Reutlingen University.

### Evaluation strategy

Our system idea is based on three assertions. Firstly, we assert that situation awareness can be outsourced from CAS and contextual information can be provided through an interface by an independent SRS. Therefore, we evaluate the fulfillment of the requirements of CAS from Table 1 to be able to act context-aware, using the middleware. Secondly, we assert that the interface can be specified using the SDC standard series. We evaluate this by verifying whether the constraints of the standard parts, introduced in “Specification and implementation of the middleware” section, have been respected. And thirdly, we assert that by implementing a middleware, contextual information can be easily provided by an SRS and obtained by CAS. For this, we analyze the required amount of development steps and lines of code.

The three evaluation steps are examined during a proof of concept study by integrating the middleware within the SRS [3] and *OR-Pad* [2] project and demonstrating its functionality in this specific use case. The middleware was tested using seven orthopedical interventions of the *OR-Pad*: hip replacement, hip replacement revision, femoral osteosynthesis, lateral partial knee prosthesis, lag screw osteosynthesis reposition, and radial head arthroplasty reposition. For preparation, available information, like preoperative images or reports, were assigned to the surgical phases of the interventions within the *OR-Pad*. In the intraoperative mode, the *OR-Pad* system was waiting for new contextual information of the SRS to provide the assigned information according to the surgical phase as well as the progress in time. During the study, we checked the provided and obtained information for correctness during each of the seven use cases.

## Results

### Specification of the interface

**Fig. 2:**
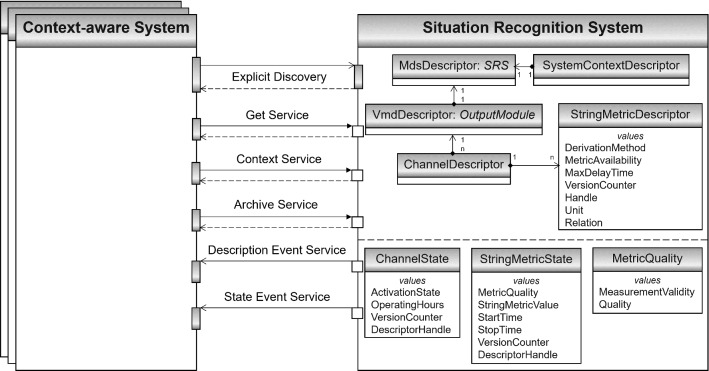
Interface architecture specified by relevant SDC objects and services. Only an excerpt of the SRS MDIB is illustrated

The SRS is defined as an SDC *Service Provider* [29], which is connected to CAS as SDC *Service Consumers* [29], as illustrated in Fig. 2. The connection must be established from the CAS using explicit discovery [33]. The CAS use the services of the SRS to obtain contextual information.

#### Data model

Contextual information needs to be stored in the *Medical Device Information Base* (MDIB) of the SRS. For MDIB modeling, only a subset of the [29] capabilities is relevant. The SRS as a *Medical Device* (with *MdDescription* and *MdState*) consists of a lean MDIB with one *Medical Device System* (MDS) and one *Virtual Medical Device* (VMD) with descriptive and stateful parts as presented in Fig. 2. Contextual information is modeled using *String Metrics*, grouped in content-related *Channels*. A metric for timing values would be advantageous but currently does not exist in the [29] data model.

The *String Metric Descriptor* specifies which kind of contextual information is provided, e.g., a surgical phase or the RSD. The current values are stored in the corresponding *String Metric State*, e.g., “Closing” as the current phase. Additional attributes are suitable for better integrating the contextual information in the individual use cases of the CAS. Dependencies between contextual information are modeled using *Relations* between the *String Metrics*, e.g., a contains-relation if a surgical phase contains subordinate surgical steps. The specified MDIB elements are listed in Table 2.

**Table 2:** Specification of the SRS’ MDIB

Element	Intended use	Example
**Channel descriptor**		
> Code	Specifies the meaning of the channel semantically unambiguously using the SDC nomenclature [32]	Functional context [30] (local extension)
> Handle	Uniquely identifies the MIDB element	CHAN_FC
**String metric descriptor**		
> Code	Specifies the meaning of the contextual information entity semantically unambiguously using the SDC nomenclature [32]	Surgical phase (local extension)
> Derivation method	Specifies whether the contextual information entity is acquired automatically or via manual input	Automatically
> Handle	Uniquely identifies the MIDB element	FC_SP
> Max delay time	Specifies the average duration from the determination of the contextual information entity to its provision in the MDIB	500 ms
> Metric availability	Specifies whether the contextual information entity is continuously or intermittently available	Continuously
> **Relation**	Specifies a dependency to another String Metric Descriptor	
>> Code	Specifies the meaning of the relation semantically unambiguously using the SDC nomenclature [32]	Contains (local extension)
>> Entries	Referencing the associated String Metric Descriptor Handles	FC_SS (surgical step)
>> Kind	Specifies the relationship to the associated metrics	Effect on containment tree entries
> Unit	Assigns a measurement unit from the SDC nomenclature [32] to the metric	Dimensionless
**Channel state**		
> Activation state	Expresses whether the channel provides valid metrics, or if all metrics are currently invalid	On
> Operating hours	Represents how long the channel provides valid metrics	2
**String metric state**		
> **Metric quality**	Express the representativeness of the current value of the contextual information entity	
>> Measurement validity	Indicates whether the value of the metric is valid or should currently not be used (e.g., if the SRS could not estimate a value)	Valid
>> Quality	Percentage of how confident the estimation of the contextual information value is	0.97
> Start time	Timestamp since when the current value of the contextual information entity has been provided	1640863190706 (December 30, 2021, 11:19:50)
> Stop time	Timestamp until when the value of the contextual information entity was valid or had been replaced by a new value	
> String metric value	Represents the current value of the specific contextual information entity	Implantation of prosthetic stem

Only an excerpt of the MDIB is listed, with elements that are relevant to provide contextual information

IEEE Standards Association [29, 34] requires that the SDC nomenclature [32] shall be used for machine-interpretable encoding of MDIB elements. The nomenclature currently does not contain codes for intraoperative contextual information. Thus, the nomenclature is extended as required in [32] and listed in Table 3.

**Table 3:** Local extension of the SDC nomenclature to encode contextual information

Systematic name	Description	Partition::Code
Situation Recognition System|Functional Context	Channel of an SRS containing metrics that offer information about the functional context according to [30]	1::61443
Situation recognition system|Procedure-related context	Channel of an SRS containing metrics that offer information about the function context according to [30]	1::61444
Functional context|Surgical phase	Current surgical phase of the intervention	2::61440
Functional context|Surgical step	Current surgical step specifying a Surgical Phase	2::61441
Functional context|Surgical activity	Current surgical activity specifying a Surgical Step	2::61442
Functional context|Relation|Contains	A granular lower-level entity of the functional context contains a granular higher entity	2::61443
Procedure-related context|Remaining surgery duration	Estimated remaining duration of the intervention	2::61444
Procedure-related context|Delay	Calculated delay of the intervention depending on the RSD	2::61445

The private areas of the nomenclature partition 1 (*Device Nomenclature*) and partition 2 (*Metrics*) were used as exemplary listed for the *OR-Pad* use case

#### Service model

The SRS recognizes contextual information and provides it in the MDIB as described above. The behavior of the SRS can be assigned to the *Medical Class A Safety Classification* [29], whereby the information provided may be used in clinical functions of the CAS, but not solely determines diagnostic or therapeutic decisions. The contextual information in the MDIB needs to be accessible for CAS. SDC designed the *Medical Devices Communication Profile for Web Services* (MDPWS) for this purpose [33]. The associated service model [29] defines the Get service as mandatory, whose operations can be used to request specific contextual information entities from the SRS. The Context service enables the orchestration of the SRS and CAS based on the *System Context* in the respective MDIBs. The Archive service shall be provided by an SRS to access previous MDIB states. This allows reconstructing the course of an intervention, e.g., for automatic documentation. For notifying CAS about context changes, the SRS offers the Description Event and the State Event service. During an intervention, the descriptive part of the MDIB shall not be modified. The temporary unavailability of contextual information entities can be represented via the *Measurement Validity* of the *Metric Quality* without changing the MDIB structure of the SRS. If all metrics in a *Channel* are invalid at some time, the *Activation State* of the *Channel* should be on standby. If CAS subscribed for changes of these MDIB entities, they will be notified automatically about this availability change. The specified services of the SRS are illustrated in Fig. 2.

### Implementation of the middleware

#### Generic implementation

The descriptive part of the SRS’ MDIB is imported as an XML file representing the contextual information that can be recognized by the SRS and shall be provided in a standard-conform way using the *sdcLib* functionality.

**Fig. 3:**
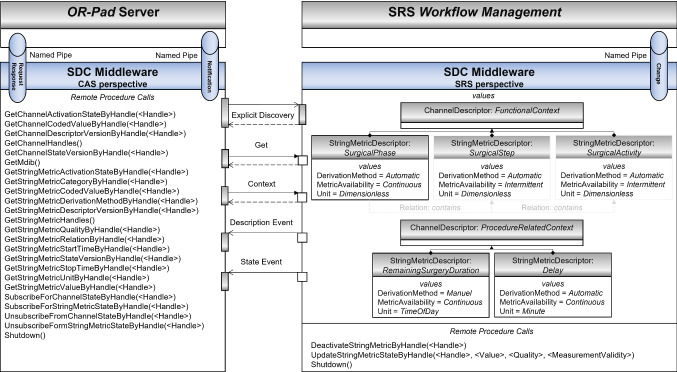
Applied interface specification for the *OR-Pad* use case resulting in a middleware architecture. Only an excerpt of the SRS MDIB is illustrated. The RPCs are accessible via the named pipes and transformed in MDPWS operations within the implemented middleware

To avoid adaptation of the SRS and CAS to the *sdcLib* syntax, RPCs were implemented to use the SDC interface within individual programs encapsulating the SDC complexity. Our implementation hides tasks such as connection establishment and MDIB maintenance. The RPCs are initiated via named pipes and transformed by the implemented middleware into MDPWS operations, based on the *sdcLib*. The middleware is executed on the SRS or CAS machines and follows the specified SDC interface as modeled in Fig. 3. The results of the RPCs, e.g., the requested contextual information, are returned via the named pipes to the individual programs. Subscriptions to the SRS can be established with the implemented RPCs. Received notifications are passed from the *sdcLib* functions to a CAS via a separate notification pipe. Changes of contextual information performed by the SRS are also triggered with RPCs through a named pipe. By using this inter-process communication between individual programs and the middleware, any SRS and CAS can exchange and receive contextual information from individual systems in a standard-conform way.

#### Integration in the OR-Pad use case

The SRS [3] implements the top-down approach according to [26], and thereby, surgical phases are continuously available while providing contextual information on a low granularity level. The *Functional Context* [30] could be specialized with surgical steps and more detailed surgical activities if the *Situation Recognition* [3] provides this differentiated view on the intraoperative situation. This conditional availability is modeled in Fig. 3, using our SDC-conformant specification. The defined contains-*Relation* is used to explicitly express these hierarchical dependencies in the MDIB of the SRS. Temporal values of the *Procedure-related Context* [30] channel, like the RSD, are provided as strings referring to the time protocol specified in the *Clock Descriptor* of the MDIB.

If the SRS has detected a new value of a contextual information entity, the Python-based *Workflow Management* [3] initiates the update of the corresponding metric. The RPCs of the SRS middleware component are used to primarily update the value of the corresponding metric and secondary set the associated attributes of the metrics, representing the current *Metric Quality*. The JavaScript-based *OR-Pad* [2] can request or subscribe contextual information in the SRS’ MDIB using the RPCs of the CAS middleware component, as modeled in Fig. 3. In our case, the *OR-Pad* simply uses the *SubscribeForStringMetricStateByHandle* RPC to subscribe to the IORC_FC_SP and IORC_PRC_D metric. If new information is retrieved via the notification pipe, the *OR-Pad* displays these in the user interface. Depending on the surgical phase, the *OR-Pad* searches for the assigned information and displays this beneath the displayed phase to make the information accessible with one click.

### Evaluation

The exchange of contextual information between the SRS and the *OR-Pad* is enabled via the implemented SDC middleware. The SRS provided the most probable recognized surgical phase, RSD, and calculated delay via updating the SDC metrics on phase change. At runtime, the *OR-Pad* subscribes to the surgical phase and delay metric and receives all changes. The retrieved surgical phase and delay are visualized depending on the estimations of the SRS. Context-relevant information that was pre-assigned to the surgical phase within the *OR-Pad* is provided to the user. A simulation of a CAS subscribed in parallel to the SRS to demonstrate that multiple CAS can be served at the same time.

The middleware provides continuous access to contextual information, collected by a central SRS and represented in the specified MDIB (R03). All other requirements (R04-R07) of the interaction category could be met as well. In the risk management category, R08–R10 are fulfilled by persisting the loose coupling between the SRS and CAS. Fault tolerance (R11) is also met since no misbehavior through missing contextual information is assumed, because CAS are informed about the availability and quality of the information entities in the MDIB via the specified attributes. Semantic interoperability (R02) cannot be achieved because the current version of the SDC nomenclature [32] does not encode intraoperative contextual information. Syntactic interoperability (R01) is lost by using the RPCs but is achieved if the plain SDC protocol is used.

Considering the standard-compliant specification of the interface, the data and service model is directly derived from the SDC standard parts [29, 32–34] and fulfills all mandatory constraints. The implementation is based on the *sdcLib*, without inconsistent customizations. The planned Archive service is currently not provided by the *sdcLib*.

The integration and usage of the middleware in the SRS and *OR-Pad* programs require only two major steps: During development, provided code snippets in Python, NodeJS, and C++ are to be copy-pasted into the system’s project. Afterward, the RPCs (listed in Fig. 3) can be used directly within the own code. Moreover, the MDIB may be adapted according to the SRS’ contextual information. The lines of code hardly depend on how many RPCs the software wants to use and if there is any pre- or post-processing of the information. For runtime usage, the middleware needs to be started first and be running during the whole usage.

## Discussion

### System goals

The presented specification and implementation of the SDC middleware is an important step toward context awareness in the OR. This allows an SRS to provide contextual information via the SDC standardized communication protocol. CAS can access this contextual information using SDC services to provide context-aware assistance.

One advantage of this separation of concerns is that the middleware and also the SRS do not depend on any context-aware use cases. In contrast to the approach of [22], a central WfMS does not decide which assistance functions are triggered and when. Due to the loose coupling to the SRS, these assistance functions are unknown to the SRS and are purely controlled by the CAS. However, unlike [21] and following the approach of [23], a central component exists in which the recognition complexity is aggregated. The SRS itself acts as an independent system, only providing contextual information to other systems. The CAS themselves are responsible for subscribing to desired information and adapting their behavior according to it. It should be noted that the SRS is the single point of failure in this system architecture and CAS and must have appropriate fallback strategies if contextual information cannot be provided or is not sufficient enough concerning the recognized quality. By specifying and implementing the middleware, the standardized security and patient safety constraints are applied [34]. All requirements concerning risk management are fulfilled, and the system goal of controllable exchange of contextual information (SG4) is achieved.

Our middleware encapsulates the complexity of establishing and maintaining an *sdcLib*-based interface. RPCs can be used in the individual programs of CAS or an SRS to access contextual information or update these in the MDIB. This encapsulation allows the developers to easily integrate the SDC interface in their systems as proven for the *OR-Pad* use case. This middleware-based architecture achieves the system goal of device-independent exchange of contextual information (SG2). The RPCs are transformed in an SDC-conform representation within the implemented middleware. The comprehensive data and service model of the SDC standard series enables specification of the configurable exchange of contextual information (SG3) to support individual CAS and the varying recognition capabilities of an SRS. The specification of the MDIB elements and services to access contextual information by an SRS can be seen as prototypic SDC *Device Specializations*.

By locally extending the nomenclature with codes for intraoperative contextual information, it is not possible to achieve cross-institutional and cross-device interoperability. Our extension is derived from the multi-perspective model of surgical situations for the context-aware OR in [30]. We aim at integrating the extension in the SDC standardization process. SPM ontologies [39] may improve the semantic and clear annotation of the contextual metrics and provision of a common structuring of the SRS’ MDIB. With these known semantics, medical device manufacturers can implement assistance functions that depend on these defined contextual information entities. If this contextual information is provided in an OR using the standardized interface with known semantics, the devices can access the SRS and automatically interpret the needed contextual information to react context-aware. However, Burgert et al. [40] emphasizes that due to the high inter-process and inter-clinical variability of surgical procedures, surgical information cannot be fully encoded in a standardized manner. In future work, we will address the challenge of local variability of contextual information to achieve the system goal of semantic interoperability (SG1).

### Applicability and transferability

In our evaluation, we have shown principal applicability along with the *OR-Pad* use case, only representing some of the capabilities of our middleware. We verified the related assertions in three evaluation steps during this proof of concept study. In the following, we discuss the validity of our results according to [41].

Internal validity [41] considers the influence of study conditions on the results. The evaluation was conducted by the developers, well knowing all system components, and, therefore, the integration of the middleware was quite easy. Other researchers may need to first get into the different RPCs that can be used and identify, how to use them in their systems. Furthermore, the MDIB of the SRS needs to be configured. This is mitigated by providing code snippets and comprehensive documentation.

The transferability of the results is argued based on external validity [41]. The concept was affected by our used SRS and *OR-Pad* as use case scenario. The evaluation was conducted in the research OR of Reutlingen University. Transfer to other research or clinical environments was not tested, but the middleware was kept generalizable to be used within other systems. Due to the standardization and encapsulation, the results are transferable to other use cases, while using the middleware in the intended manner. No matter what kind of SRS or CAS uses the middleware, the RPCs are independent of the software which uses them. Furthermore, our configuration of the generic middleware implemented for the *OR-Pad* use case can still be extended to cover more contextual information, such as the used instrument and the position of the surgeon, as it can be provided by SRS and be useful for other CAS.

Finally, the conclusion validity [41] indicates whether correct conclusions can be derived from the conducted study*.* We initially defined three assertions with individual evaluation steps for quantitative assessment. These assertions contain the fundamental aspects of our system idea. The assessment was performed once after the specification and implementation of the middleware. The evaluation steps provide successful quantitative results that allow the assertions to be accepted, as presented in “Evaluation” section. During the evaluation, we critically revised the information that the SRS sent as well as the information that was received by the *OR-Pad*. No contextual information has been lost or wrong data have been transferred. The reliability of the middleware and, therefore, of the evaluation results is given. The reliability of the contextual information of the SRS is not controllable by the middleware itself.

## Conclusion

We presented an approach that uses the current version of the SDC standard series to provide contextual information to unknown CAS. Our idea focuses on a middleware for loose coupling with an intervention-independent SRS. We showed that our middleware solves limiting factors that currently prevent context awareness in the OR. The applicability is verified using the *OR-Pad* as an exemplary CAS. The standardized modeling and exchange of contextual information enable vendor-independent context awareness. We specified the SDC data and service model for this purpose. Encapsulating the SDC complexity in an RPC-based middleware allows the exchange of contextual information with low effort. Furthermore, we identified the missing encoding of intraoperative contextual information in the current version of the SDC nomenclature. The semantically clear encoding is essential for automatically interpreting the contextual information by the CAS and enabling the proposed loose coupling with an independent SRS. The integration of a suitable SPM ontology and dealing with the variability of contextual information will be addressed in further work to achieve semantic interoperability with the presented middleware.
